# Single-Session No-Touch Hysteroscopic Mechanical Resection for Cesarean Scar Pregnancy: A Novel Primary Treatment Approach

**DOI:** 10.3390/diagnostics15233030

**Published:** 2025-11-28

**Authors:** Cihan Bademkiran, Kevser Arkan, Mehmet Yaman, Ihsan Bagli, Mehmet Obut, Mesut Bala, Mesut Ali Haliscelik, Muhammed Hanifi Bademkiran, Pelin Bademkiran

**Affiliations:** 1 Department of Obstetrics and Gynecology, Diyarbakir Gazi Yasargil Research and Training Hospital, 21010 Diyarbakir, Turkey; dr.mehmetyaman@gmail.com (M.Y.); ihsanbagli@gmail.com (I.B.); mesut.bala@gmail.com (M.B.); 2 Division of Gynecologic Oncology, Department of Obstetrics and Gynecology, Diyarbakir Gazi Yasargil Research and Training Hospital, 21010 Diyarbakir, Turkey; kevser.toprak1989@gmail.com (K.A.); mesuthaliscelik@hotmail.com (M.A.H.); 3 Division of Perinatology, Department of Obstetrics and Gynecology, Memorial Diyarbakir Hospital, 21070 Diyarbakir, Turkey; drmehmetobut@gmail.com; 4 Department of Obstetrics and Gynecology, Faculty of Medicine, Gaziantep University, 27410 Gaziantep, Turkey; hanifibademkiran@hotmail.com; 5 English Program, Faculty of Medicine, Biruni University, 34015 Istanbul, Turkey; pelinbademkiran@hotmail.com

**Keywords:** cesarean scar pregnancy, hysteroscopy, ectopic pregnancy, minimally invasive surgery, uterine niche, isthmocele

## Abstract

**Background/Objective**: Cesarean scar pregnancy (CSP) represents a challenging and potentially life-threatening form of ectopic pregnancy. This study aims to assess the feasibility, safety, and clinical efficacy of employing the hysteroscopic mechanical tissue removal system as a primary treatment modality for CSP. **Methods**: This retrospective cohort study included 53 patients diagnosed with CSP who underwent primary hysteroscopic resection at a tertiary care center. The surgical procedure was performed by prioritizing the “no-touch” vaginoscopic approach, which avoids instrumentation. Success rates, operation time, time to negative serum β-hCG, complications, and differences between the anatomical types of CSP (Type 1 vs. Type 2) were analyzed. **Results**: Primary hysteroscopic treatment was successful in 51 of 53 patients (96.2%). For the entire cohort, the median operative time was 7 min (range: 2–30), and the median interval to β-hCG negativization was 11 days (range: 6–45). The overall major complication rate was 3.8% (*n* = 2). One case was deemed unsuccessful due to conversion to laparotomy following uterine perforation during cervical dilation. Another patient, diagnosed with persistent trophoblastic disease requiring methotrexate (MTX) therapy, was also considered a treatment failure. Operative time was significantly longer in patients with Type II CSP compared with Type I (median 9 min vs. 5 min; *p* = 0.0004). **Conclusions**: Hysteroscopic mechanical tissue removal as a primary treatment for cesarean scar pregnancy represents an effective and safe “one-step” approach, characterized by a high success rate, rapid β-hCG resolution, and a low incidence of complications. This fertility-preserving, minimally invasive technique may be considered a primary treatment option for hemodynamically stable patients with CSP, provided that appropriate patient selection is undertaken and sufficient surgical expertise is available.

## 1. Introduction

The global rise in cesarean delivery rates has been accompanied by an increasing prevalence of long-term complications associated with this surgical procedure [1,2]. Among these, cesarean scar pregnancy (CSP), a rare but potentially life-threatening form of ectopic pregnancy, represents one of the most challenging entities to diagnose and manage [3]. CSP is defined by implantation of the gestational sac within the myometrium and fibrotic tissue at the site of a previous cesarean section scar [4]. If not identified promptly and managed appropriately, CSP may lead to devastating sequelae, including uterine rupture, massive hemorrhage, bladder injury, or the need for emergency hysterectomy, all of which carry significant morbidity and may compromise future fertility [5,6].

Despite increasing recognition, there is still no universally accepted consensus on the optimal management of CSP [7]. Therapeutic strategies described in the literature include systemic or local methotrexate (MTX) administration, dilatation and curettage (D&C) often performed following uterine artery embolization (UAE), and surgical approaches such as laparoscopic or laparotomic resection [8,9]. However, these modalities are associated with important limitations. MTX therapy, particularly in cases with high initial β-hCG levels or fetal cardiac activity, demonstrates reduced efficacy and necessitates prolonged surveillance [10,11]. D&C, a “blind” procedure, is associated with incomplete evacuation, excessive hemorrhage at the scar site, and an increased risk of uterine perforation [12,13]. UAE, while effective for hemostasis, entails additional invasive intervention, increased costs, and potential deleterious effects on ovarian reserve and subsequent fertility [14].

In this context, hysteroscopy has emerged as a promising, fertility-preserving, minimally invasive modality that provides real-time visualization of the gestational sac [15,16]. By enabling targeted resection under direct vision, hysteroscopy facilitates complete removal of trophoblastic tissue while minimizing injury to the surrounding healthy endometrium and myometrium [17,18]. Nevertheless, current evidence indicates that hysteroscopy has predominantly been utilized as a secondary or “rescue” intervention following failed primary treatments such as MTX or UAE, or for clearance of residual tissue after D&C [19,20].

To date, no published studies have reported the direct use of a hysteroscopic mechanical tissue removal system as the primary, single-session treatment for CSP, applied immediately after diagnosis without adjunctive therapies. To our knowledge, few reports have evaluated primary single-session hysteroscopic management using mechanical tissue removal in CSP. The present work represents one of the largest single-center series employing a standardized no-touch vaginoscopic technique with a dedicated mechanical system, and it details a reproducible operative protocol [21,22]. By specifying device parameters, entry strategy, and hemostatic measures without electrosurgery, this study aims to support safe adoption and external reproducibility in routine practice. The present study addresses this gap by introducing, for the first time, the exclusive use of a mechanical tissue removal hysteroscopic system as a “single-step treatment” strategy for CSP. Furthermore, it provides one of the most comprehensive case series to describe in detail the “no-touch” vaginoscopic technique. The objective of this study is to evaluate the feasibility, safety, and clinical effectiveness of this fertility-preserving, single-session approach in a cohort of 53 patients.

## 2. Material and Methods

### 2.1. Study Design and Ethics Committee Approval

This retrospective cohort study was conducted in the Obstetrics and Gynecology Clinic of Diyarbakır Gazi Yaşargil Training and Research Hospital after obtaining approval from the hospital’s Ethics Committee (Approval No: 194; Date: 2 October 2024). Within the scope of the study, the medical records of patients who were diagnosed with cesarean scar pregnancy (CSP) and underwent primary hysteroscopic treatment between June 2023 and June 2025 were retrospectively reviewed.

### 2.2. Patient Population and Selection Criteria

During the study period, a total of 53 patients who met the eligibility criteria were included in the final analysis. Inclusion criteria were a diagnosis of CSP confirmed by transvaginal ultrasonography (TVUS) according to the criteria described by Timor-Tritsch et al. [5], hemodynamic stability, a desire for future fertility, and having undergone hysteroscopic resection as the primary treatment. Patients were excluded if they presented with hemodynamic instability or suspected uterine rupture, wished to continue the pregnancy, had contraindications to sedation anesthesia, had previously undergone any alternative treatment for CSP, or if hysteroscopy revealed no gestational tissue within the uterine cavity despite sonographic evidence of CSP. Diagnosis of cesarean scar pregnancy (CSP) was based on ultrasound findings of a gestational sac or trophoblastic tissue implanted within the previous cesarean scar, regardless of the presence or absence of an embryo or fetal cardiac activity. Therefore, all patients meeting these imaging criteria were included, without subgroup exclusion for embryonic or anembryonic presentation. Gestational age (in days) at diagnosis was recorded for each case to explore its potential relationship with operative difficulty and complication risk. Fetal cardiac activity (present/absent) was documented descriptively at baseline.

### 2.3. Surgical Procedure

**Figure 1:**
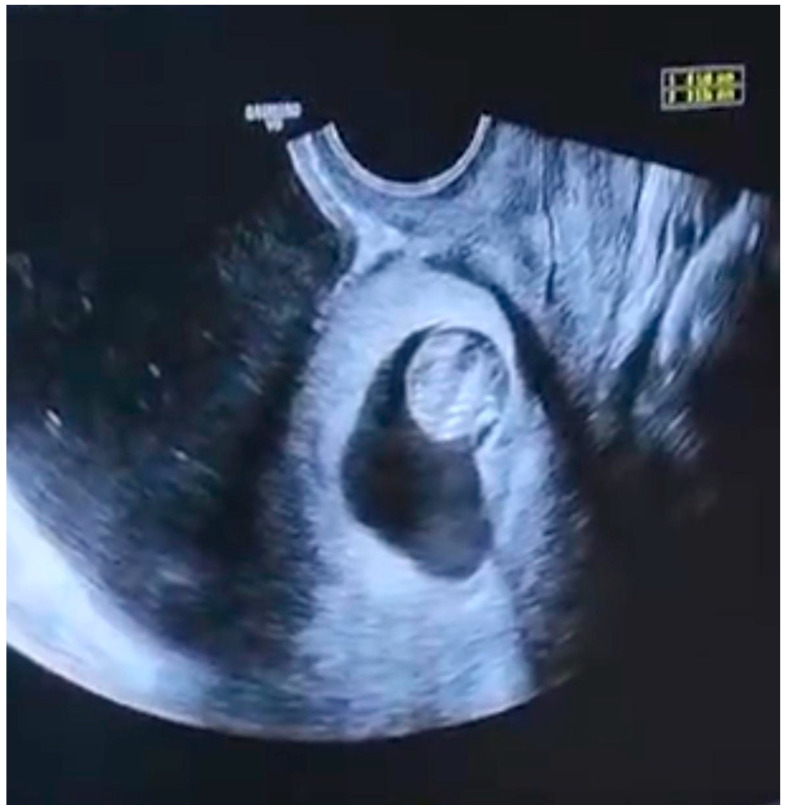
Preoperative transvaginal ultrasound image showing a cesarean scar pregnancy (CSP) sac implanted within the lower uterine segment (Type II CSP according to Timor-Tritsch classification). The scale bar indicates 10 mm.

Preoperative Preparation: All patients received 400 mcg of vaginal misoprostol approximately 8 h before the procedure to facilitate cervical ripening. Prior to surgery, a standard TVUS was performed to assess the size and location of the gestational sac, residual myometrial thickness (RMT), and the anatomical type of CSP (Figure 1). Outcomes analyzed included success rates, operative time, time to serum β-hCG resolution, complications, and differences between CSP anatomical types. CSP types were defined according to the Timor-Tritsch and Monteagudo classification: Type I (endogenic, with the gestational sac growing toward the uterine cavity) and Type II (exogenic, with implantation deep into the myometrium or toward the bladder). Although alternative terminologies (e.g., Type 0–Type 1) have been proposed, we adopted the classic Type I/II categorization to maintain consistency with established literature.

Anesthesia and Surgical Equipment: All procedures were performed by a single experienced surgeon under sedation anesthesia in the dorsal lithotomy position. For uterine cavity distension, 0.9% isotonic sodium chloride (normal saline) solution was used, with intrauterine pressure maintained within the range of 100–120 mmHg. The surgical system employed was the Medtronic TruClear™ Hysteroscopic Tissue Removal System (7.25 mm in diameter; Minneapolis, MN, USA).

All procedures were performed by a single experienced surgeon under sedation anesthesia in the dorsal lithotomy position. Sedation was standardized using a continuous propofol infusion (1–3 mg/kg/h) with supplemental midazolam (1–2 mg) as needed. For uterine cavity distension, 0.9% isotonic sodium chloride (normal saline) was used, and intrauterine pressure was maintained at 100–120 mmHg with an automatic pressure-controlled pump. Cervical preparation with 400 µg of vaginal misoprostol was routinely administered approximately 8 h before surgery unless contraindicated.

The TruClear™ Hysteroscopic Tissue Removal System (outer sheath 7.25 mm; Medtronic, Minneapolis, MN, USA) was employed in all cases. A rotary blade (model: TruClear Elite Mini, Medtronic) was operated at a cutting speed of 1500–2000 rpm with a suction flow rate of approximately 500–800 mL/min. Real-time transabdominal ultrasonography was used during the initial phase of the study to guide resection and was later limited to end of procedure confirmation of complete uterine evacuation The no-touch vaginoscopic approach avoided the use of a speculum, tenaculum, or cervical dilators in all patients.

Evolution of Surgical Technique: Over the course of the study, the surgical approach evolved based on accumulating experience. In the initial cases, cervical dilatation with Hegar dilators was used; however, this technique was abandoned after a uterine perforation occurred in one patient. In subsequent cases, to eliminate the risk of perforation associated with instrumentation, a vaginoscopic “no-touch” approach was adopted, avoiding the use of a speculum, tenaculum, or dilators, and allowing direct entry into the uterine cavity. Similarly, while real-time transabdominal ultrasound guidance was initially employed to enhance the safety of resection, with increasing surgical experience, it was reserved for the end of the procedure, solely to confirm complete evacuation of the uterine cavity.

**Figure 2:**
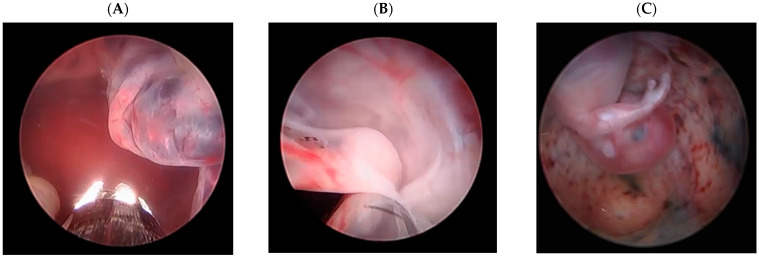
(**A**) Preoperative hysteroscopic view of the CSP tissue at the cesarean scar. (**B**) Postoperative view after complete mechanical resection. (**C**) Visualization of gestational sac contents before resection. The scale bars indicate magnification (5 mm).

Resection and Hemostasis: After entering the uterine cavity with the hysteroscope, the cesarean scar pregnancy (CSP) tissue at the level of the internal os was visualized. Using the mechanical tissue removal system, trophoblastic tissue was systematically resected until the underlying whitish fibrotic scar tissue was clearly exposed (Figure 2). Since the system lacked coagulation capability, active bleeding was controlled by temporarily increasing the intrauterine distension pressure to achieve a tamponade effect. The hysteroscope was then advanced closer to the CSP tissue to restore image clarity. Once all trophoblastic tissue had been removed, hemostasis was confirmed. Uterine integrity was assessed hysteroscopically before the procedure was concluded, and all excised tissue was sent for histopathological confirmation (Figure 1).

Fluid Management: The amount of distension fluid used and recovered during the procedure was meticulously recorded. Patients were closely monitored for signs of fluid overload, such as hypervolemia or pulmonary edema, which may result from excessive absorption of isotonic solution. Mean fluid deficit was 500 mL (range 150–2000 mL), with a maximum intrauterine pressure of 120 mmHg. No electrolyte imbalance or clinical signs of fluid overload were observed postoperatively.

Definition of Success and Complications: Treatment success was defined as complete removal of the gestational tissue in a single session, accompanied by a decline in serum β-hCG levels to <5 mIU/mL within 30 days postoperatively. Treatment success was defined as complete removal of gestational tissue in a single session without the need for additional surgical or medical therapy, accompanied by biochemical resolution (serum β-hCG decline to <5 mIU/mL) on follow-up. Time to β-hCG negativization was analyzed as a separate outcome measure. Failure was defined as the need for additional surgical intervention (laparoscopy/laparotomy) or medical treatment (methotrexate), or failure of β-hCG to normalize within 30 days. Major complications evaluated included uterine perforation, massive hemorrhage, bladder injury, and fluid overload syndrome. Major complications were predefined as uterine perforation, massive hemorrhage requiring transfusion or surgical conversion, bladder injury, and fluid overload; all other events were classified as minor.

### 2.4. Data Collection and Postoperative Follow-Up

Demographic and clinical data were retrieved from patients’ medical records. Collected variables included maternal age, parity, number of previous cesarean deliveries, gestational age, preoperative and postoperative hemoglobin levels, and baseline β-hCG values. One patient with a complication remained hospitalized for 3 days, whereas the others were discharged after a mean observation period of 8 h (range, 4–16 h). Following discharge, patients were monitored on a weekly basis until biochemical resolution (serum β-hCG < 5 IU/L), and a transvaginal ultrasound (TVUS) was performed at postoperative week 1 to assess uterine cavity status.

A step-by-step demonstration of the standardized no-touch hysteroscopic resection technique, including entry, tissue removal, and hemostasis, is provided in Supplementary Video S1.

### 2.5. Statistical Analysis

Statistical analyses were performed using IBM SPSS Statistics for Windows, version 25.0 (IBM Corp., Armonk, NY, USA). Continuous data were assessed for normality using the Shapiro–Wilk test. Descriptive statistics for continuous variables were expressed as mean ± standard deviation or median (minimum-maximum), as appropriate, and categorical variables were presented as frequency (*n*) and percentage (%). Between-group comparisons were conducted using the independent-samples *t*-test or the Mann–Whitney *U* test for continuous variables, and the chi-square test or Fisher’s exact test for categorical variables, as appropriate. All analyses were two-tailed, and exact *p*-values were reported. Key proportions, including the primary success and complication rates, were presented with 95% confidence intervals calculated using the Wilson method. The time to β-hCG negativization was analyzed using Kaplan–Meier survival curves stratified by CSP type (Type I vs. Type II) and compared using the log-rank test. A *p*-value < 0.05 was considered statistically significant.

## 3. Results

### 3.1. General Demographic and Clinical Characteristics of the Participants

A total of 53 patients were included in the study, of whom 23 (43.4%) had Type I CSP and 30 (56.6%) had Type II CSP. The baseline characteristics of the participants are summarized in Table 1. Regarding presenting symptoms, 35 patients (66.0%) were asymptomatic, while 18 patients (34.0%) presented with vaginal bleeding.

The mean age of the cohort was 35.13 ± 6.44 years. The median (range) values were 4 (2–7) for gravida, 3 (1–5) for parity, and 2 (1–4) for the number of prior cesarean deliveries. The mean preoperative and postoperative hemoglobin (HGB) levels were 12.01 ± 1.26 g/dL and 11.23 ± 1.26 g/dL, respectively. The median preoperative β-hCG level was 8790 mIU/mL (range: 1088–78,320), the median time to β-hCG resolution was 11 days (range: 6–45), and the median operative time was 7 min (range: 2–30). Postoperative TVUS evaluation confirmed complete removal of gestational tissue in all patients, with no residual pregnancy material detected.

### 3.2. Treatment Success and Complications

At 12-month follow-up, treatment was successful 51 of 53 patients (96.2%; 95% CI 87.0–99.0). The median time to β-hCG negativization was 11 days (range: 6–45). A total of two cases (3.8%) were considered treatment failures.

Kaplan–Meier curves for time to β-hCG < 5 mIU/mL by CSP type (Type I vs. Type II) are shown in Supplementary Figure S1.

All intraoperative and postoperative complications are summarized in Table 2. The fundal perforation observed during no-touch entry was recorded as a complication; however, as the resection was completed successfully in the same session without hemodynamic compromise, the case was counted as a treatment success.

Major complications occurred in 2 of 53 patients (3.8%; 95% CI 0.5–13.0):

In one patient (1.9%), uterine perforation occurred during cervical dilation with a Hegar dilator. The procedure could not be continued, laparotomy was performed to repair the perforation, and the scar pregnancy tissue was surgically removed. This case was classified as a treatment failure.

In another patient (1.9%), plateauing of postoperative β-hCG levels led to a diagnosis of persistent trophoblastic disease. This case was also considered a treatment failure and was successfully managed with a multidose MTX protocol.

In addition, after adopting the “no-touch” technique, one patient experienced a fundal perforation due to sudden advancement of the hysteroscope while passing through the internal os. Since this did not affect hemodynamic stability or visualization, CSP resection was completed in the same session, and the patient was managed conservatively. This case was recorded as a complication but counted as a successful treatment.

Table 2 summarizes the intraoperative and postoperative complications observed during hysteroscopic mechanical resection of cesarean scar pregnancy. Major complications were defined as uterine perforation, massive hemorrhage requiring transfusion or surgical conversion, bladder injury, or fluid overload. Minor complications included all other events not requiring additional surgical or medical intervention. The fundal perforation encountered during no-touch entry was recorded as a complication; however, since the resection was completed successfully in the same session without hemodynamic compromise, this case was considered a treatment success.

### 3.3. Correlation Between Gestational Age and Operative Parameters

Gestational age at diagnosis showed a strong positive correlation with operative time (ρ = 0.728, *p* < 0.001), β-hCG negativization time (ρ = 0.498, *p* < 0.001), and fluid amount (ρ = 0.644, *p* < 0.001), while a significant negative correlation was observed with myometrial thickness (ρ = −0.424, *p* = 0.002) (Table 3). These findings indicate that procedures performed at more advanced gestational ages required slightly longer operative durations and greater distension volumes, although complication rates remained unaffected. No significant differences in operative time or complication rates were observed between embryonic and anembryonic (fetal cardiac activity absent) cases.

Figure S1 was considered statistically significant. GA = gestational age; β-hCG = beta-human chorionic gonadotropin.

Surgical outcomes differed between Type 1 and Type 2 CSPs, with findings suggesting that Type 2 cases require more meticulous surgical management. The perioperative parameters comparing Type I and Type II cases are summarized in Table 4.

Table 4 Comparison of continuous clinical and surgical parameters between cesarean scar pregnancy (CSP) Type I and Type II groups. Data are presented as mean ± standard deviation or median (minimum-maximum), as appropriate.

## 4. Discussion

This study represents the first report in the literature to directly utilize a hysteroscopic mechanical tissue removal system under transvaginal ultrasonography guidance for the primary treatment of cesarean scar pregnancy (CSP), while also describing in detail the “no-touch” vaginoscopic approach that avoids uterine manipulation. The observed primary success rate of 96.2% (51 out of 53 patients) highlights this minimally invasive technique as a strong and safe option capable of providing “one-step definitive treatment” [23].

Various treatment modalities for CSP have been reported in the literature. Systemic or local methotrexate (MTX) is among the most frequently used methods, with reported success rates of 65–80%; however, the slow decline of β-hCG levels and the need for additional surgical intervention represent major disadvantages [3,24]. Dilatation and curettage (D&C) alone, being a “blind” procedure, shows lower success rates (60–70%) and residual tissue or re-intervention rates of up to 40% [4,12,25]. Uterine artery embolization (UAE) achieves success rates of approximately 75–85% and is effective in hemorrhage control; however, it is rarely curative on its own and is most often combined with MTX or hysteroscopy [26,27]. Consequently, the most commonly adopted strategies in the literature are combined approaches (MTX + hysteroscopy, D&C + hysteroscopy, UAE + hysteroscopy), which can increase success rates to 85–95%. Nonetheless, these methods are associated with two-step treatment, prolonged β-hCG normalization, higher costs, and extended hospitalization [7,15,28,29].

Complete removal of trophoblastic tissue is the cornerstone of successful CSP management [30], and this is enabled by the precision of hysteroscopy under direct visualization. Another critical advantage of our method is the rapid normalization of serum β-hCG. In our cohort, the median normalization time was 11 days, eliminating the anxiety-provoking waiting period of several weeks often seen with systemic MTX therapy [10,31]. Furthermore, this “one-step treatment” paradigm eliminates the additional costs and hospital visits associated with multi-step approaches, such as UAE or MTX followed by hysteroscopy [29].

In contrast, our study achieved a 96.2% success rate, short operative times (median 7 min), rapid β-hCG normalization (median 11 days), and a low major complication rate (3.8%) with primary one step hysteroscopic mechanical resection. These outcomes demonstrate superior efficacy and faster recovery compared with single-modality approaches such as MTX, D&C, or UAE, while offering success rates comparable to combined modalities but with the advantages of being one-step, shorter in duration, and minimally invasive. Therefore, mechanical resection may be considered a strong alternative in hemodynamically stable CSP cases due to its effectiveness and safety profile.

An important finding of our study was the evolution of the surgical technique through experience. The uterine perforation encountered during cervical dilatation with a Hegar dilator, which necessitated laparotomy, highlighted the fragility of the uterus in CSP [32]. This experience prompted us to adopt a safer “no-touch” vaginoscopic entry technique, which eliminated the risk of instrumentation-related perforation. With this refined approach, our major complication rate remained as low as 3.8% (2/53). This compares favorably with higher rates of hemorrhage and perforation reported with traditional methods such as D&C, further supporting the safety of the technique [25,33].

Our findings also demonstrated that the anatomical location of CSP (Type 1 vs. Type 2) influences surgical outcomes. Previous studies have reported that Type 2 CSPs, which are embedded deeper into the myometrium, present greater technical challenges [34,35]. In our series, the significantly longer operative times in Type 2 cases support this observation, suggesting that Type 2 CSPs require more meticulous surgical planning.

Another method employed for hysteroscopic CSP treatment is bipolar energy resection. However, bipolar resection carries the risk of thermal damage and, particularly in larger gestational sacs, necessitates repeated insertion and withdrawal of the instrument to remove tissue fragments. This can prolong operative time and complicate fluid management, thereby impairing visualization. In contrast, the mechanical tissue removal system used in our study prevents thermal injury by performing “cold cuts” and simultaneously aspirates resected tissue, maintaining continuous clear visualization. This advantage allowed rapid (median 7 min) and safe completion of procedures even in more advanced gestational ages (median 52 days) in our cohort [36,37].

Although gestational age correlated positively with operative duration and distension requirements, these differences did not translate into higher complication rates, suggesting that standardized technique and surgeon experience mitigate the effect of gestational maturity on procedural difficulty.

This study has certain limitations, including its retrospective and single-center design. The fact that all procedures were performed by a single experienced surgeon may limit the generalizability of the results, although it ensured technical standardization. Moreover, mid-term outcomes such as menstrual recovery, niche integrity, and subsequent fertility were not systematically assessed in this cohort and represent an additional limitation. Prospective longitudinal follow-up is warranted to evaluate reproductive and uterine outcomes after this procedure. Nevertheless, the strengths of this study lie in presenting one of the largest series focusing on primary hysteroscopic CSP treatment and in detailing the evolution of the surgical technique.

In conclusion, the use of a hysteroscopic mechanical tissue removal system as a primary treatment represents a highly effective, safe, and minimally invasive option for CSP. This approach ensures rapid β-hCG decline, enhances patient comfort, and provides a more efficient process compared with multi-step treatments. In particular, the “no-touch” vaginoscopic technique, which avoids instrumentation, plays a critical role in improving procedural safety. When applied with appropriate case selection and adherence to a standardized technique, it offers a rapid biochemical resolution, short recovery, and a low complication profile. These findings suggest that hysteroscopic mechanical tissue removal may serve as a valuable first-line option for selected cases of cesarean scar pregnancy in centers experienced with minimally invasive surgery.

## Figures and Tables

**Table 1:** Demographic and Clinical Characteristics of the Patients.

Parameters	Value
Uterine Niche Type, *n* (%)	
Type 1 (Endogenic)	23 (43.4)
Type 2 (Exogenic)	30 (56.6)
Presenting Symptom, *n* (%)	
Asymptomatic (Control)	35 (66.0)
Vaginal Bleeding	18 (34.0)
Treatment Outcome, *n* (%)	
Successful	51 (96.2)
Unsuccessful	2 (3.8)
Fetal Cardiac Activity (FCA), *n* (%)	
Absent	13 (24.5)
Present	26 (49.1)
Unspecified/Other *	14 (26.4)
Continuous Variables	Mean ± SD or Median (Min–Max)
Age (years)	35.13 ± 6.44
Gravida	4 (2–7)
Parity	3 (1–5)
Abortion	0 (0–4)
Number of Previous Cesarean Deliveries	2 (1–4)
Gestational Age (days)	52 ± 9.91
Preoperative Hemoglobin (g/dL)	12.01 ± 1.26
Postoperative Hemoglobin (g/dL)	11.23 ± 1.26
Preoperative β-hCG (mIU/mL)	8790 (1088–78,320)
Time to β-hCG Negativization (days)	11 (6–45)
Residual Myometrial Thickness (mm)	2 (0.5–3.7)
Operation Time (minutes)	7 (2–30)
Blood Transfusion (units)	0 (0–2)

*: FKA = fetal cardiac activity; “Unspecified/Other” includes cases without definitive embryonic cardiac assessment or anembryonic gestations recorded as non-viable on baseline imaging. SD = Standard Deviation; FCA = Fetal Cardiac Activity; β-hCG = Beta-human Chorionic Gonadotropin. Note: Values are expressed as mean ± SD, median (min–max), or *n* (%).

**Table 2:** Summary of intraoperative and postoperative complications.

Event	Classification	*n* (%)	Management	Outcome
Uterine perforation during cervical dilatation	Major	1 (1.9%)	Laparotomy and surgical repair	Treatment failure
Fundal perforation during no-touch entry	Minor	1 (1.9%)	Conservative management (observation)	Treatment success
Fluid overload or electrolyte imbalance	- (none observed)	0	-	-

**Table 3:** Correlation between gestational age and operative parameters.

Parameter	Correlation Coefficient (ρ)	*p*-Value
Operative time (minutes)	0.728	<0.001
β-hCG negativization time (days)	0.498	<0.001
Fluid amount (mL)	0.644	<0.001
Myometrial thickness (mm)	−0.424	0.002

Legend: Table 3 compares the Spearman correlation analysis results between gestational age (weeks) and selected operative or anatomical parameters. Significant positive correlations were observed between gestational age and operative time, β-hCG negativization time, and fluid amount, while a significant negative correlation was found with myometrial thickness.

**Table 4:** Comparison of Continuous Variables According to Cesarean Scar Pregnancy Type (Type I vs. Type II).

Parameter	Type I Mean ± SD/Median (Min–Max)	Type II Mean ± SD/Median (Min–Max)	*p*-Value
Age (years) *	35.04 ± 7.43	35.20 ± 5.70	0.931
Gravida **	3 (2–7)	4 (2–7)	0.138
Parity **	3 (1–5)	3 (1–4)	0.623
Abortion **	0 (0–1)	0 (0–4)	0.292
Living children **	2 (1–5)	3 (1–4)	0.384
Spontaneous vaginal delivery (SVD) **	0 (0–3)	0 (0–2)	0.252
Previous cesarean section (C/S) **	2 (1–4)	3 (1–4)	0.249
Gestational age (weeks) *	48.39 ± 9.82	54.77 ± 9.21	**0.019**
Preoperative hemoglobin (g/dL) *	12.10 ± 1.33	11.94 ± 1.22	0.652
Postoperative hemoglobin (g/dL) *	11.49 ± 1.24	11.04 ± 1.25	0.195
Preoperative β-hCG (mIU/mL) **	6320 (3102–32,546)	11,126.5 (1088–78,320)	0.055
Postoperative β-hCG (mIU/mL) **	1320 (986–11,700)	2255 (1100–27,230)	**0.045**
β-hCG negativization time (days) **	10 (6–17)	12 (7–60)	0.095
Myometrial thickness (mm) **	2.40 (1.4–3.7)	1.55 (0.5–2.8)	**<0.001**
Operative time (minutes) **	5 (2–18)	8.5 (4–30)	**<0.001**
Fluid volume (mL) **	3400 (2000–12,000)	6350 (3000–72,021)	**<0.001**
Fluid loss (mL) **	350 (150–1212)	710 (250–2000)	**<0.001**
Blood transfusion (units) **	0 (0–0)	0 (0–2)	0.381

* Independent samples t-test was used for normally distributed variables. ** Mann–Whitney U test was applied for non-normally distributed variables. Bold *p*-values indicate statistical significance (*p* < 0.05).

## Data Availability

The datasets generated and analyzed during the current study are not publicly available due to patient confidentiality but are available from the corresponding author on reasonable request. An anonymized surgical video demonstrating the procedure is publicly available as Supplementary Video S1.

## Supplementary Materials

The following supporting information can be downloaded at: https://www.mdpi.com/article/10.3390/diagnostics15233030/s1, Figure S1. Kaplan-Meier curves showing time to β-hCG negativization by CSP type (Type I and Type II); Supplementary Video S1. No-touch vaginoscopic hysteroscopic mechanical resection for cesarean scar pregnancy. The file has been fully anonymized, with no audio and no patient identifiers.

## Abbreviations

β-hCG	Beta-human chorionic gonadotropin
CSP	Cesarean scar pregnancy
D&C	Dilatation and curettage
HGB	Hemoglobin
MTX	Methotrexate
RMT	Residual myometrial thickness
TVUS	Transvaginal ultrasonography
UAE	Uterine artery embolization
