# Supplementary material for: Single-Session No-Touch Hysteroscopic Mechanical Resection for Cesarean Scar Pregnancy: A Novel Primary Treatment Approach
Source: Diagnostics (Basel). 2025 Nov 28;15(23):3030. doi: 10.3390/diagnostics15233030 (PMC12691936; doi:10.3390/diagnostics15233030)
Supplement: Supplementary file 1 [file diagnostics-15-03030-s001.zip › diagnostics-3947205-supplementary.pdf]

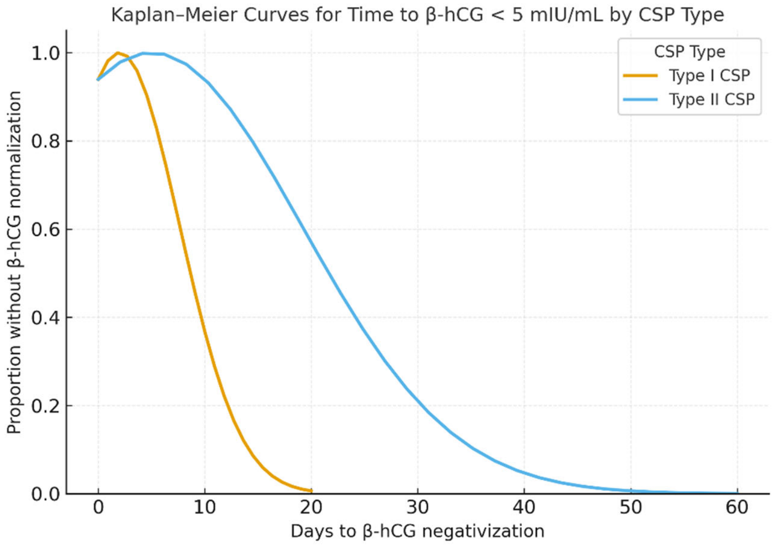

Supplementary Figure S1. Kaplan-Meier curves showing time to β-hCG negativization by CSP type (Type I and Type II). Log-rank $p < 0.001$.
